# Social Science Collaboration with Environmental Health

**DOI:** 10.1289/ehp.1409283

**Published:** 2015-05-12

**Authors:** Elizabeth Hoover, Mia Renauld, Michael R. Edelstein, Phil Brown

**Affiliations:** 1 American Studies and Ethnic Studies, Brown University, Providence, Rhode Island, USA; 2 Department of Sociology and Anthropology, Northeastern University, Boston, Massachusetts, USA; 3 School of Social Sciences and Human Services, Ramapo College of New Jersey, Mahwah, New Jersey, USA

## Abstract

**Background:**

Social science research has been central in documenting and analyzing community discovery of environmental exposure and consequential processes. Collaboration with environmental health science through team projects has advanced and improved our understanding of environmental health and justice.

**Objective:**

We sought to identify diverse methods and topics in which social scientists have expanded environmental health understandings at multiple levels, to examine how transdisciplinary environmental health research fosters better science, and to learn how these partnerships have been able to flourish because of the support from National Institute of Environmental Health Sciences (NIEHS).

**Methods:**

We analyzed various types of social science research to investigate how social science contributes to environmental health. We also examined NIEHS programs that foster social science. In addition, we developed a case study of a community-based participation research project in Akwesasne in order to demonstrate how social science has enhanced environmental health science.

**Results:**

Social science has informed environmental health science through ethnographic studies of contaminated communities, analysis of spatial distribution of environmental injustice, psychological experience of contamination, social construction of risk and risk perception, and social impacts of disasters. Social science–environmental health team science has altered the way scientists traditionally explore exposure by pressing for cumulative exposure approaches and providing research data for policy applications.

**Conclusions:**

A transdisciplinary approach for environmental health practice has emerged that engages the social sciences to paint a full picture of the consequences of contamination so that policy makers, regulators, public health officials, and other stakeholders can better ameliorate impacts and prevent future exposure.

**Citation:**

Hoover E, Renauld M, Edelstein MR, Brown P. 2015. Social science collaboration with environmental health. Environ Health Perspect 123:1100–1106; http://dx.doi.org/10.1289/ehp.1409283

## Introduction

The work of social scientists has improved our understanding of the diverse impacts of such human-caused events as leaking hazardous waste sites, chemical explosions, and oil and gas spills, as well as of human-exacerbated natural disasters. Social scientists have conducted ethnographic case studies of communities suffering environmental catastrophe in order to place environmental health impacts into the contexts in which they are experienced. This includes psychological health, impacts due to the loss of physical health, the difficulty of proving causality in health impact, and community mobilization. Because contamination extends beyond the physical into sociocultural patterns of everyday life, social scientists supplement environmental health research by providing a more complete picture of the impacts on individuals and communities.

Here we discuss a recent innovation in social science work on environmental contamination: the emergent, boundary-crossing effort to integrate social science with environmental health practice. This new approach moves beyond pure research to intervention, reflecting increasing collaboration between social scientists and environmental health scientists to measure exposures, press for cumulative exposure to be addressed, and prepare research data as the basis for health policy. Contemporary research on environmental inequalities is being moved out of isolated disciplinary silos to actively engage across disciplines; to work directly with affected communities to investigate exposures and resulting health effects that have already occurred; and to influence environmental policy to mitigate primary (actual hazard) and secondary (individual, community, and societal) impacts of past exposures and to prevent new exposures from occurring. In this way, the social scientist becomes an actor in events rather than a mere observer.

A fundamental catalyst for social science–environmental health collaboration has been the National Institute of Environmental Health Sciences’ (NIEHS) Community-based Participatory Research (CBPR) and Environmental Justice Programs and its more recent umbrella program, Partnerships in Environment Public Health (PEPH) (NIEHS 2012a). NIEHS’s support of environmental justice and CBPR has contributed to the study of communities affected by environmental hazards. This incorporation of social science to enhance community-level understanding of contamination has also benefited from the community engagement cores that are part of center grants: Superfund Research Program, Children’s Environmental Health Centers, Environmental Health Core Centers, and Breast Cancer and the Environment Research Centers. Conferences have evolved from these collaborations, exploring case studies and demonstrating the importance of team science. Two examples are the 2012 Superfund Research Program workshop at Brown University and the 2011 Environmental Reproductive Health Symposium organized by Native Americans, Alaska Natives, and others (Hoover et al. 2012). This commentary originated at NIEHS’s 2013 Environmental Health Disparities and Environmental Justice Workshop, which included scholars from sociology, anthropology, psychology, and environmental health (NIEHS 2013).

In this commentary, we begin with the “social science *of* environmental health” by discussing the contribution of detailed social science case studies of contaminated communities. We then discuss key issues in social science research on environmental health and justice, and identify emerging issues and new directions in research, communication, capacity building, training, and evaluation. These features shape the boundary crossing to “social science *with* environmental health,” exemplified by a case study of research projects in Akwesasne that was one of the first to incorporate CPBR and social science in environmental health.

*Processes of discovery in contaminated communities*. Over the past three decades, social scientists have conducted in-depth studies of how laypeople discover and act on environmental problems, typically in the face of a crisis or discovery that has placed the “contaminated community” (Edelstein 1988, 2004) in the public eye. Social scientists understood early on that many cases of contamination are discovered by laypeople, not by experts. Nevertheless, the public continues to expect government to actively monitor the environment to protect public health, yet public pressure from affected citizens or community organizations is often necessary for recognition and remediation of environmental exposure.

Much of the data collected about environmental impacts come through core regulatory programs, for example, periodic testing by permit holders required for compliance with the Clean Air Act of 2004 or the Clean Water Act of 1972. Regulatory agencies often lack the resources or a clear mandate to review the data, which could result in a lack of enforcement. U.S. environmental law includes provisions aimed to empower citizens as a corrective for such flaws. These include publication of hazards data through “right-to-know” provisions of the Superfund Amendments and Reauthorization Act of 1986, Title III, known as the Emergency Planning and Community Right-to-Know Act (EPCRA; Cho et al. 2008). Citizens can now review these public data directly online, as they can for other key environmental statutes, or request additional information through the Freedom of Information Act. Environmental laws also provide for extensive rights to public comment and input, and, in some statutes, if violations are found that have not been subject to government enforcement, the public—as “citizens’ attorney generals”—can bring the violator to court.

Despite these opportunities, there is generally an awareness gap. Most citizens remain unaware of hazards unless pushed to investigate by some incident or pattern of problems. Unless nongovernmental organizations (NGOs) or citizens are diligent, the majority of environmental data are never examined for their place-based implications. Moreover, key environmental data for the health of communities may never be collected if there is no regulatory driver, and cumulative impacts are generally not addressed. Local governments, closer to the problems, may be compromised in their ability to act due to conflicting interests.

When scientists study local environmental exposures, many do not report findings to the study participants. Social science–environmental health collaborations have worked to address this by increasing the amount of report back to research participants, leading to more sound data and the development of more democratic public policies that advance environmental literacy (Brody et al. 2014).

*Social science case studies of environmental health and justice*. Laypeople’s role as the typical discoverers of hazards, clusters, or environmental health threats, and the impacts of such discoveries, have been documented through a rich legacy of ethnographic social science research and within newer social science–environmental health partnerships.

Ethnographic contributions. The earliest ethnographies detailing environmental health and justice cases are rooted in narrative tales of the experiences of residents from discovery to action.

In 1972 at Buffalo Creek, a poorly constructed, inadequately maintained dam broke, causing a lake of coal mining slurry to sweep down the Kentucky hollow, destroying a poor Appalachian community (Erikson 1976). The flood razed hundreds of homes, killed 125 people, injured many others, and left psychological scars. Sociologist Kai Erikson was asked by attorneys representing survivors to draft a report, and his findings connected individual trauma with collective loss of communality (Erikson 1976). Later, his report became the first book-length community study of a human-caused environmental disaster. Erikson’s innovations placed human-made disaster into the cultural, social, and historical context of the community; addressed the individual mental health and physical health outcomes of affected individuals within the cumulative community effects; and demonstrated that social science can work to help affected people.

Adeline Gordon Levine’s (1982) *Love Canal: Science, Politics, and People* recounted the story of a residential neighborhood of Niagara Falls that was developed adjacent to a buried toxic waste site, a fact uncovered by community residents who fought a 2-year battle for relocation, paving the way for creation of the U.S. Environmental Protection Agency (EPA) Superfund program. Levine and her students conducted observations and interviews, attended public meetings and events, and maintained a constant presence in the community to fully document the many impacts of contamination. A significant contribution was the analysis of the role of scientists who choose to work alongside community activists, developing mutually beneficial research questions and analytic strategies.

Michael Edelstein, a social and environmental psychologist, began examining the impact on communities and individuals both from existing and proposed hazardous sites in *Contaminated Communities: The Social and Psychological Impacts of Residential Toxic Exposure* [Edelstein 1988; revised with new title as *Contaminated Communities: Coping with Residential Toxic Exposure* (Edelstein 2004)]. Here he examined social and psychological impacts of water contamination in Legler, New Jersey, contrasting it with other cases. What he described as “environmental turbulence” occurs as normal life is replaced by sometimes desperate adaption. People initially try to cope, using their own personal and family resources, and, if that fails, they turn to their trusted social networks, which may also be inadequate. They then call upon institutional networks (i.e., government officials), from whom they expect help. Inevitably, they become dependent on expert researchers and scientists to verify both the toxic threat and its causal link to symptoms. This dependency is disabling, particularly when these institutions fail to meet victims’ needs, to adequately mitigate the contamination, or to inform residents of environmental safety in their own homes. Edelstein’s concept of “environmental stigma” addressed contamination as a threat to identity. His notion of the “inversion of home” explored how the safe haven of one’s home is transformed into a constant source of danger and fear. He also examined the “disabling” loss of control and distrust associated with both environmental exposure and the social response to it, and the “debilitating” loss of health optimism, yet also charted the “enabling” dynamics that allowed communities to coalesce to act proactively.

Lee Clarke’s (1989) *Acceptable Risk? Making Decisions in a Toxic Environment* detailed hazard perceptions after the Binghamton, New York, state office building fire, focusing on the political and economic features that shaped what was purported to be a neutral approach to assessing risk. Martha Balshem’s (1993) *Cancer in the Community: Class and Medical Authority* also looked at the hazard perceptions of people in a Philadelphia, Pennsylvania, working-class neighborhood, contrasting the individual-blaming approach of the cancer hospital where she worked with the industry-blaming approach of sufferers. Kroll-Smith and Couch’s (1990) *The Real Disaster is Above Ground: A Mine Fire and Social Conflict* studied social conflicts between different groups of residents dealing with an underground mine fire in Centralia, Pennsylvania. Picou and Gill (1996) examined chronic psychological stress associated with the Exxon Valdez oil spill. Political scientist Michael R. Reich’s (1991) *Toxic Politics: Responding to Chemical Disasters* compared the Seveso, Italy, dioxin explosion, the Michigan polybrominated biphenyl (PBB) cattle-feed contamination, and the polychlorinated biphenyl (PCB) contamination of cooking oil in Japan. Reich highlighted the long duration of resolution and compensation, and the frequent lack of support from mainstream environmental groups. Robert D. Bullard’s (1990) *Dumping in Dixie: Race, Class, and Environmental Quality* was the first work to appear in the quickly exploding field of environmental justice. In this and other work, Bullard documented how systematic environmental racism leads to health inequities by excluding certain segments of the population based on race and class from environmental decision making (Bullard 1990, 1993). In what became a fast-growing literature, other social scientists have provided analyses of environmental justice organizing efforts that highlight community voices. For example, Roberts and Toffolon-Weiss (2001) described the processes of social and political organizing as African-American and Native-American communities battled a chemical plant, a nuclear facility, an oilfield dump, and a landfill in Louisiana.

In addition, social scientists have highlighted the research roles of affected residents. In *No Safe Place: Toxic Waste, Leukemia, and Community Action,* Brown and Mikkelsen (1990) conceptualized “popular epidemiology” to describe lay involvement in community health studies. The approach emphasizes concerns of access, trust, confidentiality, data sharing, researcher reflexivity, and benefits to the people and community being studied. Families in Woburn, Massachusetts, pressured state and federal agencies to investigate a cancer cluster, and they sued W.R. Grace and Beatrice Foods for contaminating municipal water wells with trichloroethylene (TCE) and perchloroethylene (PCE), which were associated with a large number of childhood leukemia cases. Residents worked with biostatisticians to conduct 5,010 interviews, covering 57% of Woburn residences via telephone. The results showed clear connections between contaminated water, leukemia, and other health outcomes. Their efforts put Woburn alongside Love Canal as a key example of community-initiated research that engages partnership between environmental health and social scientists.

Additional social scientific contributions. Social scientists who examined the demographics of communities affected by contamination identified inequalities according to race and poverty and laid the foundation for the environmental justice movement. Bullard’s (1990) earliest work was followed by extensive work on demographics of hazardous waste sites (Faber and Krieg 2002; Mohai and Saha 2007). Social scientists, especially from geography and urban planning, have integrated quantitative GIS (geographic information systems) techniques into community mapping projects (Corburn 2005; Huang and London 2012; Maantay 2002). When communities coalesce to deal with contamination, there is often a spillover to broader sustainable community development. In this realm, urban planners have been central to the environmental health aspects of transportation, land use, and food policy (Agyeman 2013). Communications studies scholars have contributed much toward understanding science communications processes and how diverse publics understand environmental health (Nisbet 2009) and have developed new models of environmental health literacy (Zoller 2012). The work of Vogel (2012) on bisphenol A (BPA) and Krimsky (2000) on endocrine-disrupting compounds demonstrates key environmental health contributions to examining and developing chemical policy. At the global level, social science–environmental health collaborations require an understanding of political ecology and economic policies (Faber 2008).

Economists have examined the relationship between environmental policies, exposure, and community action. For example, when the public is educated about exposure through federal measures, corporate firms often experience negative stock price effects from public response; this can lead them to reduce their emissions and improve their environmental performance more than other corporate firms in their industry (Cho et al. 2008; Konar and Cohen 1997).

Early psychological work on environmental contamination focused on the Three Mile Island disaster (e.g., Dohrenwend 1983; Houts et al. 1988). Similar community-scale case–control research subsequently appeared, comparing contaminated and noncontaminated places, including stress measures (Baum et. al. 1990), fear of cancer (Hallman and Wandersman 1995; Wandersman et al. 1989), and psychological dysfunction (Gibbs 1989).

Much attention has been paid to the construction of risk, viewed as an outgrowth of cognitive evaluation of the severity of consequence and likelihood of an adverse event’s occurrence (e.g., Slovic 1993). This cognitive work facilitated a disciplinary crossover into economic research examining risk cognition on issues such as radon gas (e.g., Smith and Johnson 1988). Risk research was used in conjunction with environmental stigma, for example, to describe the basis for resistance in Nevada to the siting of the Yucca Mountain Nuclear Repository (Flynn et al. 2001).

Anthropological work has also been important in examining the impacts of such disasters as the Chernobyl nuclear disaster and its impact on Saami reindeer herders in Scandanavia (Beach 1990; Stephens 1987) and nuclear waste siting (Stoffle et al. 1991).

This transdisciplinary potential detailed above was further catalyzed by federal initiatives, as we now describe.

*Facilitating the boundary crossing*. As collaborations between social science and environmental health researchers proliferated, case studies began to provide a more complete understanding of environmental exposure that depicted both individual- and community-level effects while demonstrating environmental exposure and harm within landscapes and bodies.

Environmental social science benefits from research directions at the NIEHS. NIEHS has made tremendous strides in environmental health research by incorporating social scientists. The political climate in the 1990s paved the way for rising support for government action on environmental issues, especially after the People of Color Environmental Leadership Summit (1991) and the development of the Principles of Environmental Justice (EJ) (Bullard 1993). Additionally, Kenneth Olden, a supporter of EJ and of community involvement in research, was appointed as the third director of NIEHS in 1991. By 1995, NIEHS had become the first NIH institute to create a CBPR grant initiative. New programs focused on EJ and the ethical, legal, and social implications of scientific research offered the infrastructure needed for social scientists and community groups to enter the NIEHS sphere. Annual meetings brought together grantees, creating a network in which environmental health and social science researchers learned from one another and developed additional collaborations. Eventually, social science research became a requirement for some NIEHS programs and projects, an essential step for promoting interdisciplinary environmental health research (Baron et al. 2009). NIEHS inaugurated PEPH (2014) in 2008, providing an umbrella for community engagement and research translation across its center programs.

These partnerships led to social scientists publishing in a wide array of journals such as *Environmental Health Perspectives, Environmental Science & Technology*, *American Journal of Public Health,* and *Environmental Justice*. Beyond that, scientists from different disciplines brought together through NIEHS programs held relevant conferences, such as a 2012 Superfund Research Program national office conference on the “Social, Psychological, and Economic Impacts of Superfund and other Contaminated Sites” (NIEHS 2012a). This brought together community representatives, sociologists, anthropologists, economists, NIEHS, U.S. EPA, state agencies, lawyers, and developers to explore transdisciplinary science at the intersection of psychological, cultural, economic, physical, and health considerations.

Transdisciplinary environmental health research has increased awareness of effects beyond the physical and health consequences of environmental disaster and contamination to include community empowerment, ethical practices of sharing data, and policy implications. An example is the Household Exposure Study (HES), a CBPR project that evaluated exposures to pollutants from legacy contaminants, consumer products, and local emissions (Brody et al. 2009). Silent Spring Institute, an independent research center, collaborated with academics and the EJ organization Communities for a Better Environment (http://www.cbecal.org/) to collect data in multiple communities using biomonitoring, a tool used by environmental health scientists to explore the body burden of exposure (Brody et al. 2009). Community members were engaged at every level, as participants rather than subjects, about their report-backs and their scientific understanding (Adams et al. 2011; Brown et al. 2012). The integration of social science in the HES has facilitated the development of new theories such as the “research right-to-know” (Morello-Frosch et al. 2009), “exposure experience” (Altman et al. 2008), and “politicized collective illness identity” (Brown 2007) that have redefined and restructured exposure studies as a whole, while also increasing public understanding, environmental health literacy, community empowerment, and mutual trust and respect between researchers and study communities.

Social science–environmental health transdisciplinarity also develops in CBPR projects without social scientists as formal collaborators. Projects led by environmental health scientists (Haynes et al. 2011; Wing et al. 2008) were framed around a holistic collaboration that highlighted the importance of lay discovery, a facet that social scientists had pioneered in their ethnographic studies. They also focused on the combination of both individual-level and community-level effects, and understood the interactive nature of community development and health improvement in cleanup and mitigation. Indeed, CBPR as a whole can be viewed as essentially social scientific in light of its thorough inclusion of community involvement, community and organizational capacity building, political–economic context, and the centrality of social, psychological, and economic impacts, instead of only physiological ones (Israel et al. 1998; Minkler and Wallerstein 2010). Above all, the increasingly important participation of community members and organizations is a major component of transdisciplinarity. We might even say that CBPR itself has become a social scientific approach. It has also brought social science into the policy framework, coming full circle from merely describing negative outcomes to actually assisting cleanup, mitigation, and exposure prevention.

Social scientists and environmental data. Another important trend is social scientists’ direct involvement in the collection of environmental data, combining social science and environmental science research processes. This is exemplified by Public Laboratory for Open Science and Technology (http://publiclab.org), where co-founder anthropologist and science studies scholar Sarah Wylie uses low-cost community monitoring devices to map oil spills, flood-ravaged hydro-fracturing sites, hydrogen sulfide emissions, and other environmental disasters. Helium balloons equipped with digital cameras, hydrogen sulfide detectors using photographic paper, and thermal bobs to detect water temperature increases are among the devices that are made easily accessible to communities. This enables communities to report environmental devastation that is often unknown or overlooked by regulatory agencies, and compile data that can be used to develop and advocate for policy (Wylie, in press). This growing trend in science–technology–society, also called science and technology studies (STS) encourages social sciences to be more practical and hands-on in the scientific enterprise. In addition, many EJ scholars work with various community groups to offer technical assistance and community monitoring (Conrad and Hilchey 2011; Ottinger and Cohen 2011).

*Toward a transdisciplinary approach*. What emerges from our commentary is a new transdisciplinary approach for environmental health practice that fully engages the social sciences to paint a full picture of the consequences of contamination so that policy makers, regulators, public health officials, and other stakeholders can better ameliorate impacts and prevent future exposure. These transdisciplinary collaborations replace the solo researcher with actively engaged CBPR teams though a series of negotiations and recursive interactions between disciplinary practices that bring together social scientists, environmental health scientists, and community groups and residents (Petts et al. 2008). This reflexive and iterative research process moves beyond multidisciplinarity, in which researchers maintain their respective disciplinary methods and perspectives, to a truly interdisciplinary form that fully integrates and engages with the overlaps and intersections between disciplines to ensure that all facets are investigated (Russel et al. 2008). Furthermore, these projects give communities data to fully comprehend their exposure experience, to pressure government agencies to respond to and remediate environmental harm, and to bring about policy change that is proactive and precautionary to prevent other communities from experiencing similar problems.

As demonstrated in the case study below, social and psychological collateral impacts are inextricably linked to contamination and individual and community well-being. Even when excellent environmental health research studies are conducted, social science methods are necessary to fully understand and mitigate the impact of environmental contamination.

*Akwesasne: a case study at the intersection of CBPR and transdisciplinary environmental health research*. The nearly 15 years of environmental health research conducted in the Mohawk community of Akwesasne provides a prime example of community/social science/environmental health collaboration. Research was undertaken as a partnership between the Akwesasne Mohawk organizations Akwesasne Task Force on the Environment (ATFE; http://www.northnet.org/atfe/atfe.htm) and First Environment Research Project (FERP), and the State University of New York at Albany (SUNY) (Schell and Tarbell 1998). Hoover (2010) examined the history of this project to evaluate its CBPR approach and to elaborate on how this model can influence future studies.

Akwesasne, whose territory is bounded by New York State and the Canadian provinces of Ontario and Quebec, is downstream from a number of polluting industries including General Motors (GM), Alcoa, and Reynolds Aluminum (now Alcoa East). In 1981 two sludge pits filled with PCB-contaminated waste were discovered behind GM, adjacent to the Raquette Point region of the reservation (Hoover 2013). By 1984 the entire 270-acre GM property was declared a Superfund site. Following tests done by a New York State wildlife pathologist that found high levels of PCBs in fish and aquatic wildlife, an official three-part health risk assessment was undertaken to examine contaminant levels in fish, wildlife, and breast milk (Forti et al. 1995).

The impetus for undertaking scientific research to determine community impacts came from the Mohawk themselves. Mohawk midwife Katsi Cook insisted that state and university officials work with Akwesasne as equal partners to investigate contamination levels and, later, health impacts. Akwesasne community members recognized the limits of conventional health risk assessments and therefore sought to incorporate social science research in risk assessment and management (Arquette et al. 2002). As a result, decision makers were able to supplement scientific data with a more holistic and comprehensive evaluation of impacts on health, incorporating the knowledge and experience of the at-risk population (Arquette et al. 2002). The SUNY Superfund Basic Research Program (SBRP) worked directly with the affected community to achieve this outcome throughout the 1990s and early 2000s.

With the help of their community partners, the SBRP established a connection between fish consumption and PCB levels in Mohawk women’s breast milk and in men’s blood (Carpenter et. al 2002). Fish advisories against the consumption of local fish were issued by the tribal governments and the New York State Department of Health as a protective measure, but interviews conducted with Akwesasne community members indicated that the loss of fishing affected traditional cultural and social systems and exacerbated diet-related health problems in the community (Hoover 2013). This demonstrates how the auxiliary impacts of risk avoidance recommendations such as fish advisories must be considered. Combining environmental science with social science data allowed the Saint Regis Mohawk Tribe Environment Division (2013) to issue a more nuanced, revised fish advisory. While the advisory works to prevent the consumption of fish from certain species or locations, it provides instructions on how to consume other fish in a healthy manner.

Environmental health studies conducted by the SUNY team and the Akwesasne community under a second SBRP grant focused more on specific health conditions linked to the local contamination. These projects produced more than a dozen papers demonstrating myriad health impacts linked to PCB body burden in adolescents and adults (e.g., Codru et al. 2007; Schell and Gallo 2010). In each of these projects, ATFE members collaborated with SBRP research team scientists at SUNY Albany to design the studies, and Mohawk women were trained to collect data from community members, reshaping the research process to include community members as co-producers of knowledge rather than passive subjects (Schell et al. 2007). Some scientists from the project have since gone on to conduct additional CBPR projects with indigenous communities (e.g., Carpenter et al. 2005).

This series of Akwesasne studies was one of the first examples of a large-scale CBPR project. For the Mohawk, it was the first time environmental health researchers directly reported personal data back to individuals. Mohawk participants were interested in having a social scientist explore the perceptions of these studies by both community members and scientists. In the resulting report, Hoover (2010) found that scientists cited the benefit of better quality participation by the community and the greater degree of learning about Mohawk culture, but also cited the greater amount of time the study and subsequent publications took because of continuous negotiation with their community partners. Mohawk fieldworkers appreciated the opportunity to work in the community and learn transferable skills such as phlebotomy and conducting psychological surveys. Study participants generally appreciated the scientists’ effort to provide data feedback, but had suggestions for more socially and culturally relevant means of report-back, including gathering family groups together for a more interpersonal explanation of results (Hoover 2010). These findings have been received with interest from the SUNY team for possible incorporation into future study report-back. Despite tremendous progress made by CBPR in this case study, more can be done, such as deeper incorporation of social science research to help elucidate the community’s eco-historical context and to help foster positive influences on lifestrain, lifescape, and lifestyles that mitigate contamination.

## Conclusion

This commentary demonstrates the development and importance of social science–environmental health collaboration for improving environmental health science by focusing on the multiple scales of socioenvironmental impact. These new models of environmental health investigation need to be explored, evaluated, and expanded so the field can continue to develop and refine new research approaches. It is clear that toxicology, epidemiology, exposure science, and environmental engineering cannot do the job alone in studying, remediating, and preventing contamination. Social scientists, whose mission is to understand human interaction and organization, explore how contamination comes in large part from human-caused activity and how it affects social, economic, and political aspects of everyday life beyond the physical environment and bodies.

Take, for example, the recent explosion of environmental health research on flame-retardants. As Cordner (in press) shows, the bioaccumulation, mechanisms, and health effects require a history of the chemical industry’s efforts to expand the use of flame retardants, the tobacco industry’s work to avoid fire-safe cigarettes, and the efforts of a multi-party alliance of scientists, firefighters, manufacturers, and advocates to reform flammability standards and to work on chemical bans and regulations.

Conversely, the successes of social science–environmental health collaboration can be used to rethink the potential of social science to be more policy oriented and applied, in addition to being more theoretical. The repertoire of social science skills needed to conduct this work includes ethnographic interviews and observation among communities, industry, scientists, and advocacy offices, as well as technical understanding of the relevant science.

The elements addressed in this commentary are congruent with NIEHS’ Strategic Plan (NIEHS 2012b), which emphasizes translational science; pursuing CBPR; understanding how nonchemical stressors, including socioeconomic and behavioral factors interact with other environmental exposures; emphasizing health disparities; highlighting communications and engagement; developing collaborative and integrative approaches; fostering cross-disciplinary training; expanding environmental health literacy; studying the ethical, legal, and social impacts of environmental health research; and evaluating economic impact of policies, practices, and behaviors. Although NIEHS has incorporated these team science efforts, other agencies have been less engaged, and it is important to get them committed to such work. To complement this expansion, both fields need interdisciplinary training grants and individual pre- and postdoctoral fellowships that emphasize social science–environmental health collaborations, including at nonprofit organizations. Social science–environmental health partnerships need to initiate a traveling externship, similar to the Superfund Research Program’s KC Donnelly Externship (NIEHS 2015), where young scholars and community organization members can work with others doing social science–environmental health research. Networking must proliferate through more conferences. NIEHS could broaden funding opportunities for social science research, and *Environmental Health Perspectives* could create a special issue or section on social science approaches.

Increasingly there has been less of a distinction between exposure and health effects research and community ethnography. Transdisciplinary CBPR advances environmental health sciences as a whole while increasing the public’s understanding of and participation in science, trust in the research collaboration, ability to empower and sustain community-based organizations, and policy advocacy that will help to mitigate exposure.
